# Online Videos as a Source of Physiotherapy Exercise Tutorials for Patients with Lumbar Disc Herniation—A Quality Assessment

**DOI:** 10.3390/ijerph18115815

**Published:** 2021-05-28

**Authors:** Stephan Heisinger, Dominikus Huber, Michael Paul Matzner, Timothy Hasenoehrl, Stefano Palma, Julia Sternik, Carmen Trost, Michael Treiber, Richard Crevenna, Josef Georg Grohs

**Affiliations:** 1 Department of Orthopedics and Trauma Surgery, Medical University of Vienna, 1090 Vienna, Austria; michael.matzner@meduniwien.ac.at (M.P.M.); carmen.trost@meduniwien.ac.at (C.T.); michael.treiber@meduniwien.ac.at (M.T.); josef.grohs@meduniwien.ac.at (J.G.G.); 2 Department of Physical Medicine, Rehabilitation and Occupational Medicine, Medical University of Vienna, 1090 Vienna, Austria; dominikus.huber@meduniwien.ac.at (D.H.); timothy.hasenoehrl@meduniwien.ac.at (T.H.); stefano.palma@meduniwien.ac.at (S.P.); julia.sternik@meduniwien.ac.at (J.S.); richard.crevenna@meduniwien.ac.at (R.C.)

**Keywords:** herniated disc, lumbar region, spine, physical therapy modalities, instructional film and video

## Abstract

*Background:* During the last few decades the prevalence of lumbar disc herniation has been increasing constantly, thereby imposing a significant socioeconomic burden. Physiotherapy plays a crucial role in both surgical and conservative treatment of lumbar disc herniation, consequently the current COVID-19 pandemic with concomitant lockdowns has led to a shortage of physiotherapeutical care. In the light of these recent events publicly available physiotherapy tutorials may be a useful tool to address this problem. *Aim:* The main aim of this study was to assess the quality of online physiotherapy exercise tutorials for lumbar disc herniation. *Materials & Methods:* With YouTube being a widely known and used platform we screened 240 of the most viewed videos. A total of 76 videos met the inclusion criteria and were statistically analyzed. The videos were assessed using Global Quality Score, DISCERN Score and JAMA benchmark criteria and in regard to their applicability. *Results:* They displayed a wide range of views (44,969 to 5,448,717), likes (66 to 155,079) and dislikes (6 to 2339). The videos were assessed using Global Quality Score, DISCERN Score and JAMA benchmark criteria and in regard to their applicability. Neither the number of “Views”, “Likes”, nor “Dislikes” was found to have a significant association with any of the quality measures used in this study. *Conclusion*: Overall quality grade was determined as “moderate”. Based on the data examined in this study, the use of YouTube videos as a source of therapy advice for lumbar spine disc herniation cannot be recommended universally.

## 1. Introduction

Lower-back pain (LBP) and sciatica inflict a significant socioeconomic burden nowadays as well as disabling the affected patients [1,2,3,4,5,6,7,8,9,10]. As reviewed by various authors approximately 80% of the population sustains an episode of LBP once during their lifetime, consequently incurring an annual cost of more than USD 100 billion in the United States of America [11,12,13]. Furthermore LBP, lumbosacral radicular pain and sciatica are commonly associated with lumbar disc herniation (LDH) and degenerative disc disease and frequently lead to a significant decrease in the quality of life (QoL) of affected patients [10,14,15].

Although it has been extensively studied the treatment of choice for LDH remains a controversially discussed topic [11]. Various authors have shown equivalent medium- to long-term outcomes in operatively and non-operatively treated patients, with faster pain relief in operatively treated patients, whereas other authors have demonstrated superior long-term outcomes for operatively treated patients as reported by Gugliotta et al. [11,16,17,18,19,20]. Beyond all doubt physical therapy also plays a crucial role in the rehabilitation process of operatively treated patients, but its’ role in non-operative treatment regimens is even more striking, considering that the majority of patients with LDH are treated non-operatively with a multimodal approach including adequate pain medication, patient education and physical therapy [11,21].

The current COVID-19 pandemic and concomitant restrictions such as lockdowns, etc. have led to—at least temporarily—a significant shortage of conventional physiotherapeutical care [22,23,24]. Concurrently various attempts to substitute the lack of physiotherapeutical care such as telerehabilitation, etc. have emerged [22,23,25]. Although substantial effort is put into developing the aforementioned treatment substitutes, the majority of patients do not have immediate access to them for various reasons. However, it is beyond all doubt, that the internet has become one of the most important sources for health-related and medical information [26,27]. As reported by various authors the majority of the North American population with access to the Internet uses it to obtain information on health-related issues [26,28,29]. YouTube (Alphabet, Mountain View, CA) has become one of the most popular and influential websites for sharing and watching videos on the Internet with more than 1 billion visitors monthly [26,30]. Approximately 73% of U.S. adults use YouTube [31]. However, the uploaded content does not undergo a peer-review process, accordingly the quality and scientific soundness of the content remains unclear [26]. Nevertheless, patients may use YouTube to inform themselves in regard to health-related issue and moreover may use a publicly accessible video-based physiotherapy tutorial to treat their conditions, especially in the light of the current pandemic [26,32,33]. Considering the lack of control mechanisms for the uploaded content and the diverse quality there is an obvious risk of obtaining misleading and inadequate information on health-related issues [26,34,35,36].

To our knowledge there are no studies evaluating the quality of YouTube videos providing exercises for patients with symptomatic LDH. Especially in the current situation with an ongoing COVID-19 pandemic it appears crucial to evaluate the quality of the most viewed videos to determine the potential applicability of physiotherapy tutorials on YouTube for patients with LDH.

## 2. Material and Methods

The study design was purely descriptive. Similarly to Kocyigit et al., we used the search items “lumbar disc herniation exercise”, “lumbar disc herniation rehabilitation”, “lumbar disc herniation physical therapy” and “lumbar disc herniation physiotherapy” on YouTube (www.youtube.com, accessed on 6 June 2020) and aimed to assess physiotherapy exercise tutorials for patients with lumbar disc herniation [33]. The YouTube videos were sorted according to their number of views and the 60 most viewed videos for each search item were evaluated as described by Kocyigit et al. [33]. Concludingly 240 videos in total were evaluated and off-topic videos, duplicates, videos with a language other than English or otherwise inadequate videos (e.g., poor video/audio quality) were excluded [33]. In accordance with these criteria, 76 were included in this study. The following information was recorded during screening: (1) Title of the video, (2) Universal Resource Locator, (3) number of total views, (4) number of likes, (5) number of dislikes and (6) sources of the videos. The video sources were classified as “Chiropractor”, “Health/Lifestyle Company”, “Physiotherapist”, “Physician” and “Trainer”. Categories with 2 or fewer observations were grouped as “Others”. The sources were classified by the observers based on the information obtained from the uploader’s profile.

The included 76 videos were assessed by 5 independent observers. Raters were classified as “PMR” (Physiatrist), “ORT” (Orthopedic Surgeon), “SPO” (Sport Scientist), “PHY” (Physiotherapist) and “STU” (medical student). All of the observers are fluent in spoken and written English and the healthcare professionals are well renowned experts regarding the treatment of spine patients. Three commonly used scoring and grading systems were used to evaluate the videos [26]. The included YouTube videos were assessed in regard to their quality by five independent observers based on the Global Quality Scale (GQS). The GQS is a commonly used tool to evaluate the educational quality of health-related content on the Internet ranging from 1 to 5 (1 = very poor quality; 5 = excellent quality) [33,37]. The observers assessed the videos independently from each other, in contrast to other studies no attempts to find a consensus were made if there was a discrepancy [33]. In order to assess the reliability of the included videos, the modified DISCERN tool was applied, which resembles a five-point assessment tool as reviewed by Kocyigit et al. [33,38]. It consists of five binary yes/no questions with each positive answer yielding 1 point, the maximum score is 5 [33]. Additionally, the videos were assessed using the Journal of the American Medical Association (JAMA) benchmark criteria to determine the accuracy and reliability. As recently reviewed by Springer et al., the JAMA tool is a nonspecific and objective assessment tool consisting of 4 criteria, whereas 1 point is assigned for the presence of each criterion [26]. Furthermore, the observers rated the videos on a subjective basis with a scale ranging from 1 to 5 (1 = excellent to 5 = insufficient). In analogy the observers graded the videos in regard to the technical sound and video quality on a scale from 1 to 5. Additionally, the observers determined whether advertisements were present (yes/no). To further determine the potential applicability of the included videos the observers evaluated two questions: “Does the therapy match the diagnosis?” and “Can the exercises be done at home?”. The according answers were recorded in a binary yes (=1)/no(=0) manner.

### Statistical Analysis

Data was stored and processed for further analysis in MS Excel (Microsoft Corporation, 2018. Microsoft Excel, Available at: https://office.microsoft.com/excel, accessed on 6 June 2020). Statistical Analysis was carried out in GraphPad Prism (GraphPad Prism version 9.1.0 for Windows, GraphPad Software, San Diego, CA, USA, Available at: www.graphpad.com, accessed on 6 June 2020) and SPSS (IBM Corp. Released 2020. IBM SPSS Statistics for Windows, Version 27.0. Armonk, NY, USA: IBM Corp).

Descriptive analysis included mean (metric variables) and percentiles (scores). Hypothesis testing (for difference in medians) employed Man–Whitney U tests, association was estimated via Spearman’s Rho and rater agreement using Fleiss’ Kappa. Difference in distribution was tested via modified Chi-Square tests for multiple variables.

Normality and log-normality tests via Kolmogorov–Smirnov and Shapiro–Wilk tests were conducted for all non-ordinal variables and each sub-group separately. While “Likes” and “Views” were assumed to be following a normal distribution (*p* = 0.11 and *p* = 0.24) as a whole, the variables within each group and “Duration” failed the normality-tests at the 5% level. Therefore, only non-parametric tests were employed to compare sub-groups. In all ANOVAs (both parametric and non-parametric) Bartlett’s test for homoscedasticity was checked. In several instances the assumption of homogenous variances was rejected, begging the question if these sub-groups are indeed sampled from the same population.

An alpha of 0.05 was assumed to constitute statistical significance. Where appropriate, confidence intervals are reported, also using alpha = 0.05. In all cases, two-sided testing is performed.

## 3. Results

A total of 76 videos were statistically analyzed. They displayed a wide range of views (44,969 to 5,448,717), likes (66 to 155,079) and dislikes (6 to 2339) with their percentiles displayed in Table 1. The average video lasted 386 seconds with a maximum of 1335 and a minimum of 63 seconds.

Median GQS quality over all raters was “generally poor” (GQS = 2) with a total of 6.6% “poor quality” (GQS = 1), 46.1% “generally poor”, 40.8% of “moderate quality”, 6.6% “of good quality” and 0% of excellent quality. The median Discern Reliability Score was 2 with 40.8% scoring 1, 47.4% with 2 points, 3.9% with 3 points, 2.6% with 2 points, and none with all points. The JAMA benchmark had a median of 0 with 88.2% of videos scoring 0 points, 10.5% scoring 1 and one video scoring 2 points.

**Figure 1:**
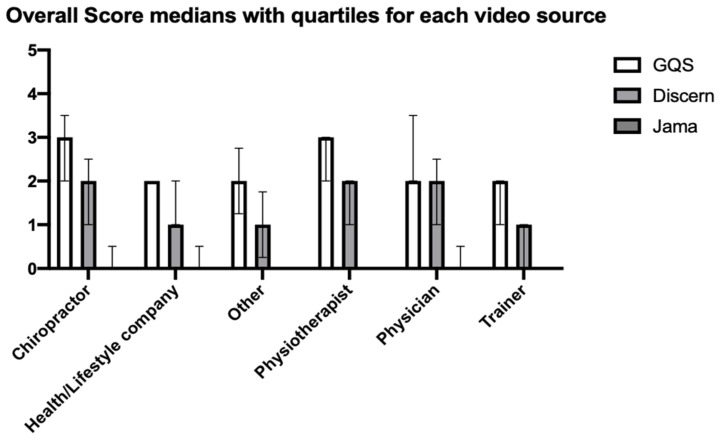
Median Score for GQS, Discern and Jama benchmarks by video source with upper and lower quartiles. Mean Ranks for GQS and Discern Score were significantly higher in Chiropractors and Physiotherapists when compared to Trainer videos.

Data showed significant differences (in a Kruskal–Wallis test) in medians of GQS (*p* = 0.001) and Discern Score (*p* = 0.002), but not JAMA benchmark (*p* = 0.425) among video sources (Figure 1). Multiple comparisons testing suggested significantly higher mean ranks for Discern Scores of “Chiropractor” vs. “Trainer” (*p* = 0.011) and “Physiotherapist” vs. “Trainer” (*p* = 0.003) as well as higher mean ranks for GQS for “Chiropractor” vs. “Trainer” (*p* = 0.003) and “Physiotherapist” vs. “Trainer” (*p* = 0.009).

**Figure 2:**
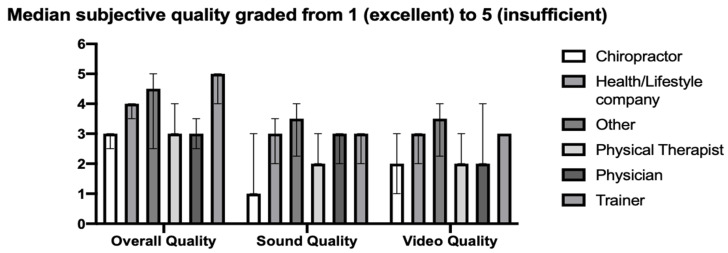
Median subjective grade for overall, video and sound quality displayed by video source including the upper and lower quartile. While no significant difference in means could be observed among video sources regarding their sound and video quality, a better overall grade was assigned to physiotherapist and chiropractor videos when compared to trainer videos.

The subjective grades (1 = excellent to 5 = insufficient) given to each video by each rater had a median of 3 for overall quality, sound quality and video quality. When grouped by video source, a Kruskal–Wallis test for difference in medians could not detect any variation with regard to video or sound quality but showed a significant variation in overall grade (*p* = 0.001). Dunn’s corrected multiple comparisons of again “Physiotherapist” vs. “Trainer” (*p* = 0.034) and “Chiropractor” vs. “Trainer” (*p* = 0.004) suggested significantly lower mean ranks of overall grade for the trainer videos (Figure 2).

**Figure A1:**
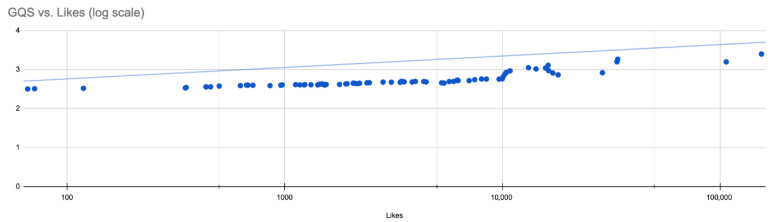
Likes were plotted on a log scale due to the typical distribution of this variable. When plotted against the GQS of the corresponding video, a positively sloped but almost horizontal line describes their relationship. This (non-)association seems to be statistically significant in a Spearman correlation (r = 0.29 [0.06; 0.49], *p* = 0.012). Note: the log-transformation of the “Likes” variable is done here for ease of viewing and does not change the outcome of (non-parametric) statistical analysis.

**Figure A2:**
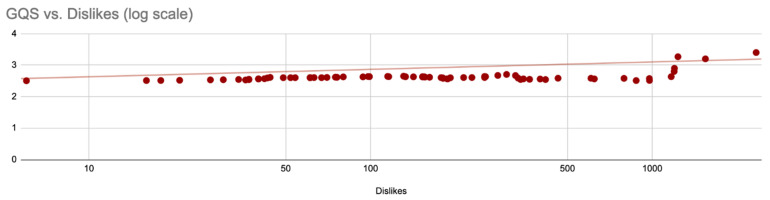
Dislikes were plotted on a log scale due to the typical distribution of this variable. When plotted against the GQS of the corresponding video, an almost horizontal line emerged. A similar picture appears for all other combinations of Likes, Dislikes and Views vs. the quality scores employed in this study.

The quality measurement tools employed in this study showed no association with “Likes”, “Dislikes” or “Views” of the evaluated videos (see Appendix A, Figure A1 and Figure A2). In particular, none of the quality scores showed a significant Spearman correlation with “Views” (GQS: *p* = 0.108, Discern: *p* = 0.121, JAMA: *p* = 0.454). Neither the Discern tool (*p* = 0.302) nor the JAMA score (*p* = 0.270) displayed significant correlation with “Likes” while the GQS yielded a significant result that there was indeed no correlation (r = 0.29 [0.06; 0.49], *p* = 0.012).

When analyzed by source of video, no particular source stood out. A Kruskal–Wallis test could not detect significant differences between the medians of different content creators regarding “Views” (*p* = 0.097), “Likes” (*p* = 0.155) or “Dislikes” (*p* = 0.365) and a modified ANOVA could not find a significant difference in mean duration (*p* = 0.364).

A comparison of median GQS via the Man–Whitney U test showed no significant preference of either the physiotherapist for videos done by physiotherapists (*p* = 0.187), nor the physiatrist or orthopedic surgeon for videos created by physicians (*p* = 0.578). Video sources classified as “Trainer” (of course rarely equivalent to a sport scientist) were graded by the sport scientist significantly worse than all other sources (Median of 2 for Trainers vs. 3 for non-Trainers, *p* = 0.003).

**Figure 3:**
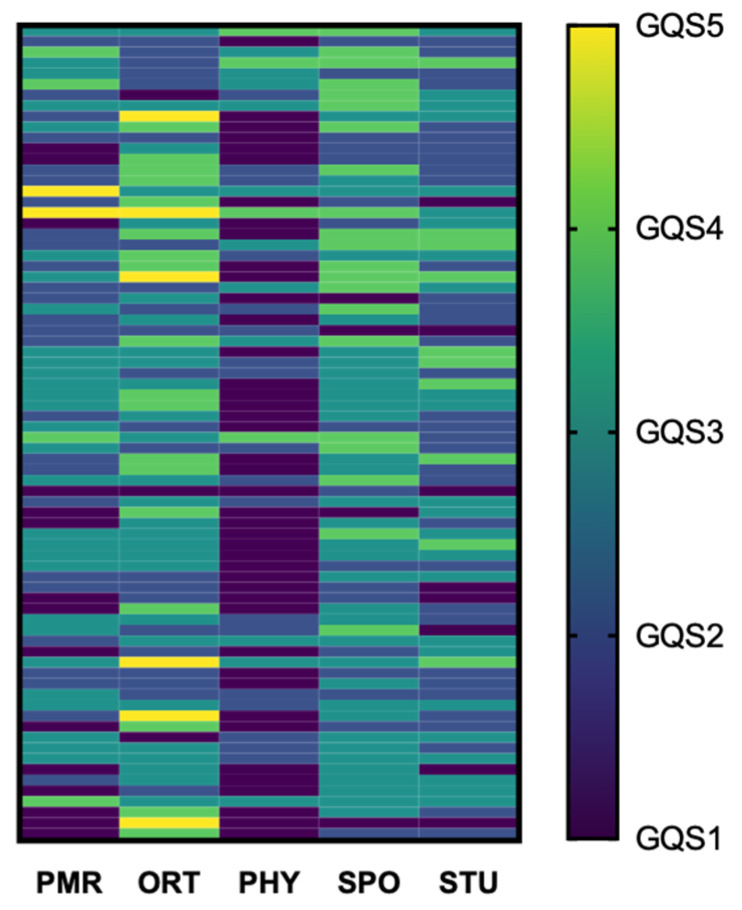
A heatmap of answers Global Quality Scale (GQS) assessments (1 = poor quality to 5 excellent quality) for each video (rows) and each rater (columns). Generally, GQS was low with a median of 2. A Kruskal–Wallis test with subsequent multiple comparisons showed significant differences between all rater’s mean ranks except for PMR vs. STU and ORT vs. SPO. Rater agreement as measured by Fleiss’ Kappa was poor (0.06).

Raters showed differences in how they scored and graded the videos. Figure 3 plots GQS (dark color for low numbers, light color for high GQS) as a scoring-pattern for all videos (rows) that differs from rater to rater (columns). If raters would agree on a certain score for a certain video the row for that video would be colored equally and a striped pattern would emerge. Figure 3 diverges from this ideal striped pattern, indicating considerable disagreement between raters. A similar image can be created for the Discern Tool.

Indeed, a Kruskal–Wallis test found significant differences in the median GQS among raters. Subsequent testing corrected via Dunn’s test showed significant differences between the mean ranks of all pairs of raters except PMR vs. STU and ORT vs. SPO.

While not matching in absolute values, some raters showed significant, albeit only moderate correlation in their GQS. PHY correlated (Spearman’s rho) moderately strong, and significantly with SPO (r = 0.50, *p* < 0.001) and PMR (r = 0.57, *p* < 0.001) and so did PMR and SPO (r = 0.47, *p* < 0.001). There was a weak, significant correlation between STU and SPO (r = 0.32, *p* = 0.004) and STU and PMR (r = 0.30, *p* = 0.008). ORT displayed no correlation with any of the raters.

The discern tool showed highly significant, (*p* < 0.001) weak to moderate (r < 0.50) correlation between all raters except PHY and SPO (*p* = 0.48) and the JAMA score was highly significantly correlated among all raters.

Despite low absolute agreement, significant correlation as measured by spearman’s rho was observed.

**Figure 4:**
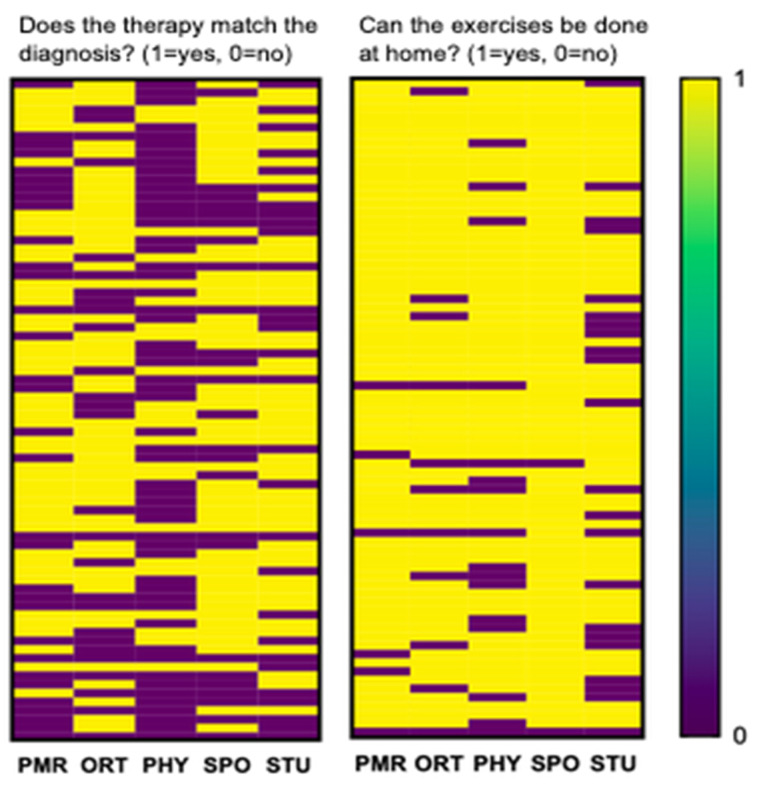
Heatmap of answers (yes = yellow, no = purple) to the questions “Does the therapy match the diagnosis?” (left panel) and “Can the exercise be performed at home?” (right panel). The heatmap displays significantly divergent answers (modified Chi-Square test *p* > 0.001) to each video (one video = one row) from each rater (one column = one rater). In absolute terms, raters agreed unanimously more often on “Home” (52 of 72 answers) than on “Match” (5 of 76 answers).

Raters were asked two questions: “Does the therapy match the diagnosis?”, which was classified as “Match” for the statistical analysis as well as “Can the exercises be done at home?” classified as “Home” with both questions coded 1 = yes and 0 = no. Figure 4 plots the answers (yes = yellow, no = purple) for each video (rows) from each rater (columns) for each of the two questions. Absolute agreement among raters was 6.6% (5 out of 76) for “Match” and 55.3% (42 out of 76) for “Home”. A Chi-square test modified for multiple variables showed a significantly different distribution among the raters (*p* < 0.001) for both questions.

Rater agreement was estimated to be poor regarding all quality scores employed in this study. Rater agreement on GQS was measured by Fleiss’ Kappa for multiple raters and estimated at 0.06 (*p* = 0.002) for all values of GQS and even lower for low values of GQS (see Appendix A, Table A1). For the Discern tool (kappa = −0.003, *p* = 0.867), JAMA score (kappa = −0.016, *p* = 0.508) and overall grades (kappa = 0.052, *p* = 0.009), raters displayed a similar agreement with a tendency of higher (but still poor) kappa-values around medium values and less agreement on the fringes.

## 4. Discussion

Beyond all doubt, the internet has become an essential and easily accessible source of information for a patient, as various authors have reviewed previously [26,32,33]. Nevertheless, it has also been shown that patients are concerned in regard to the reliability and validity of the provided information on the internet [28,29,33]. YouTube (Alphabet, Mountain View, CA, USA) is one of the most popular and influential websites on the Internet with approximately 73% of U.S. adults using YouTube [26,30,31]. However, considering that the uploaded content does not undergo a peer-review process the quality and scientific soundness of the content remain unclear [26].

Numerous previous studies have determined the overall quality of information of YouTube videos in various medical subspecialities and moreover in the field of orthopedics to be insufficient or rather poor [26,30,33,39,40,41]. Kocyigit et al. assessed the quality of ankylosing spondylitis exercise videos available on YouTube, however to our knowledge no previous study has assessed the quality of physiotherapy tutorial videos on YouTube for patients with lumbar disc herniation [33]. Hence, the evaluation of these publicly available videos appears even more crucial in the light of the ongoing pandemic.

The COVID-19 pandemic has led to demanding challenges in treating and diagnosing patients with spinal disorders such as lumbar disc herniation [22,23,25]. Meanwhile various guidelines for distance management of, e.g., spinal disorders have been published; however, the field of telemedicine still requires further research, validation and consequently improvement in order to provide the patients sufficiently [22,25,42]. As reported by Minghelli et al., the majority of physiotherapists interrupted their work in person and the overall accessibility and availability of physiotherapy has decreased during the ongoing pandemic [23].

Therefore, we hypothesized that publicly available online physiotherapy tutorials on YouTube may be a valuable source of patient education, information and treatment for a common medical condition such as lumbar disc herniation. Considering the aforementioned circumstances, it appears very likely that patients investigate the internet or more specifically online videos as a substitute for in-person physiotherapy. Although there might not be a common gold standard for the treatment of lumbar disc herniation due to various factors (e.g., neurological deficit, patient’s expectations, etc.) the crucial role of physiotherapy in both operatively and non-operatively treated patients remains undisputed [11,21].

In contrast to other studies, the videos were not assessed by professionals of one single medical/healthcare subspeciality but by a physiatrist, an orthopedic surgeon, a sport scientist, a physiotherapist and a medical student. On the one hand, this broader variety of observers offers insight into the different approaches of the various subspecialities and on the other hand supports a more objective overall assessment. The medical student served as a “medically educated but not specialized” control. Raters displayed considerable disagreement on whether a video matched the diagnosis and if it could be done at home. Less disagreement and moderate correlation could be observed for the quality scores used to evaluate the videos in this study. It could be argued that the limited interobserver reliability is a limitation of the study; however, it actually offers a more objective, broader review of the quality of the assessed videos based on the various approaches of each medical subspeciality. Nevertheless, this also emphasizes the need for further studies to determine the videos’ potential therapeutic value in relation to the experts’ opinions in order to further establish a “gold-standard” for video-based physiotherapy tutorials for patients with spinal disorders.

In order to determine whether the source of the video correlates with their quality, statistical analysis was performed accordingly. Overall, raters favored chiropractor and physiotherapist videos over trainer videos with regard to GQS, Discern Tool and Overall Quality which is generally spoken consistent with the findings of Kocyigit et al. [33].

Neither the number of “Views”, “Likes” nor “Dislikes” was found to have a significant association with any of the quality measures used in this study, which is likewise consistent with the findings in previous studies [33]. This is in fact a crucial aspect considering there was a significant number of videos with numerous views and “Likes” which on the hand were rated as poor or mediocre in regard to their quality by the observers. Moreover, it raises the question how these videos gained such popularity. As mentioned before, further studies on how patients rate the videos are needed. Additionally, other aspects such as the possibility to “buy Likes” need to be taken into consideration. There are numerous companies that offer “bought Likes” which may influence the “popularity” of the provided videos to an unknown degree. Taking this into account the value of the number of Views, Likes and Dislikes as a measurement for quality or even popularity is limited.

Median GQS of all videos was poor, overall quality grade “moderate” which coincides with the findings of other studies on the quality of YouTube videos [26,30,33,39,40,41]. Taking all of these findings into account it suggests that consumers are provided with mediocre to poor quality content and furthermore cannot rely on measures such as the number of “Views”, “Likes” nor “Dislikes”. Moreover, if patients—due to the lack of availability of in-person physiotherapy during pandemics—decide to educate themselves in regard to physiotherapy for lumbar disc herniation, they are at risk for insufficient, inadequate or misleading information. This is actually more relevant than ever given the current circumstances, considering that inadequate exercises, that may not even match the diagnosis, can severely aggravate the patients’ symptoms.

The finding that there was no significant difference in regard to the technical video and sound quality can be explained by the pre-selection where videos with poor sound and video quality were excluded.

This study has several limitations. Although various well-established scores were used to assess the quality of the videos, they rely on the observer’s subjective judgement. Additionally, the scoring in regard to technical video and sound quality was a subjective decision of each observer. Furthermore, the questions “Does the therapy match the diagnosis?” and “Can the exercises be done at home?” were assessed subjectively. As previously stated by Kocyigit et al., this study represents a single snapshot in time, which might be controversial due to the dynamic structure of YouTube [33]. The videos were screened 3 months into the pandemic and further studies will be needed to determine whether the current pandemic caused any changes in regard to quality and reliability of physiotherapy tutorials for lumbar disc herniation. Another limitation of the study is that we only searched for videos in English and search results may vary due to geographic location, internet use, etc. [33]. Moreover, another limitation of this study is that there was only one observer per subspeciality, which merely hints that the videos are rated differently by each subspeciality but does not confirm this hypothesis. Consequently, further studies will be required to determine whether the observer’s rating might differ according to his/her medical subspeciality. Furthermore, studies on how patients rate the videos in regard to quality are needed in order to gain a better understanding on which factors patients rely on to subjectively determine the quality of those videos. In several instances the assumption of homogeneity of variances was rejected, begging the question if all sub-groups were sampled from the same population. If this is the case, comparisons between groups are problematic and potentially meaningless. Although it could be reasoned that all videos share enough characteristics to be assumed to stem from the same population, the prevalence of heteroskedasticity poses a limitation to this study.

## 5. Conclusions

Telemedicine and web-based patient education have become more and more relevant during the last decade with technology evolving faster than ever. Specifically, the current COVID-19 pandemic highlights the potential benefits of telemedicine and web-based patient education. Given the immense popularity and easy accessibility of YouTube it could be used to provide patients with high quality information and tutorials on physiotherapy for lumbar disc herniation. However, based on the data examined in this study, the use of YouTube videos as a source of therapy advice for lumbar disc herniation cannot be recommended universally. Furthermore, our results highlight the need for peer-reviewed high-quality content on platforms such as YouTube as well as ongoing research in regard to the clinical applicability of video-based physiotherapy tutorials. The results of our study suggest that there is a need for high quality online physiotherapy tutorials for patients with lumbar disc herniation and moreover that those videos should be produced and validated by a board consisting of specialists from all medical subspecialities that are involved in patient treatment.

## Figures and Tables

**Table 1:** 25th, 50th and 75th percentile of views, likes and dislikes of all videos analyzed in this study.

	Median	Upper Quartile	Lower Quartile
Views	375,039	726,654	133,117
Likes	2962	8347	1232
Dislikes	153	332	55.75

## Data Availability

The data presented in this study are available on request from the corresponding author.

## Appendix A

**Table A1:** Fleiss’ Kappa estimated in order to assess rater agreement was determined to be poor in this data set both overall and for each individual value of GQS.

GQS Rating.	Fleiss’ Kappa	*p*-Value	LB 95%CI	UB 95%CI
overall	0.066	0.002	0.025	0.107
1	0.063	0.084	−0.008	0.135
2	0.051	0.159	−0.02	0.123
3	0.107	0.003	0.035	0.179
4	0.028	0.449	−0.044	0.099
5	0.042	0.249	−0.03	0.114
